# Analecta

**Published:** 1842-10-22

**Authors:** 


### ANALECTA.

*Enlarged Prostate.*—Dr. John C. Warren exhibited to the Boston Society for Medical Improvement, on the 23d of May, a specimen of enlarged prostate, of the same class, he said, with several which he exhibited to the Society some time since, and which were of much practical importance. The present specimen was taken from a gentleman 84 years of age. He had for many years had some slight obstruction in his urine. About four months since he had a more serious difficulty, which, however, was relieved. Finally he had another, when he was attended by Dr. Homans, who at first relieved him, but on the third day failed to introduce the catheter. Dr. Hayward was called in, who succeeded in drawing off his water. The day after, however, he failed, and Dr. Warren saw him. He succeeded in introducing the catheter, but drew off no water. On examination by the rectum, the parts were found very loose, and the pelvis filled with this mass of the prostate gland. He decided to let him alone, concluding that the difficulty arose from an enlargement of the middle lobe of the prostate, closing over the orifice of the urethra like a valve, and preventing the catheter from reaching the urine. On the second day after, the bladder had extended up to two fingers' breadth above the umbilicus. The bladder was punctured above the pubis, and the urine passed off very well. The second night after, the canula, by accident, was pulled out ; but it was introduced again. For several days after, the patient did perfectly well, exhibiting no symptoms indicative of any but a favourable termination. Finally, however, owing to his advanced age and circumstances connected with his previous disease, he gradually sunk and died, a result which must be considered as entirely unconnected with the puncture of the bladder.

The difficulty of passing the catheter in this case, was not owing to want of room, but to the enlargement of the third lobe of the prostate, occasioning an irregularity in the course of the urethra. It is very rare that any difficulty of this kind arises from enlargement of the lateral lobes. Dr. Warren had made many dissections of the prostate gland, and often from its minuteness was unable to discover the middle lobe, and when so large as to be discovered, it was rarely more than twice the size of a pea.—*New England Quarterly Journ. of Med. and Surg.* Oct., 1842.

*Catheter broken off in the Bladder, and Calculus formed upon it.*—The specimen was sent to the Boston Society for Medical Improvement, on the 30th of May, by Dr. HENRY TUCK, of Barnstable, Massachusetts, together with the following history of the case.

Dea. A. C. æt. 83 years, Yarmouth, a mariner in the early part of life, and farmer during the last 40 years, always enjoyed vigorous and uninterrupted good health until the beginning of 1841, when he began to experience some little difficulty in voiding his urine. He had to make considerable effort at times to relieve himself, especially when fatigued. On the 19th of May, 1841, there was retention of urine for twenty-four hours, accompanied by great pain and tumefaction in the hypogastric region. A small-sized catheter was introduced into the bladder by Dr. Oliver Ford, of this town, the attending physician, and a large quantity of water drawn off, giving immediate and complete relief. Cathartics, diuretics, tinct. of cantharides, &c., were given. Still the inability to expel voluntarily his urine continued, and the same catheter which had been long in use was passed, occasionally meeting some little obstruction from the prostate gland, for ten days, when on applying unusual force to introduce the instrument, something was perceived by the operator to give way. On withdrawing the catheter, it was found broken off at the bend, leaving the end in the urethra or bladder. A larger instrument was then passed without difficulty, and with it the piece of the broken one was distinctly felt in the bladder. Subsequent to this accident he continued his medicines, had the catheter passed twice a day for a month, and then the bladder recovered its tone, and he was able to void his urine by his own efforts. He gradually regained his health and strength, and was able to do light work on his farm until the 9th of December following, when he was seized with rigors, heat nausea, vomiting, pain in bowels, accompanied by constipation and partial retention of urine. This ceased, however, after the application of hot fomentations. During the interval between these two attacks he was obliged to micturate more frequently than usual, and his water deposited, on remaining in the vessel, a thick white, slimy sediment, which was adherent, and would rope when poured out.

On the return of his illness the second and last time, his strength was completely prostrated—there was considerable irritative fever. His water was passed involuntarily in bed. He had one or two dejections daily without cathartics. Morphine was administered to allay the pain. His mind continued unimpaired until the 7th of January, 1842, when he died.

*Post-mortem.*—On opening the abdomen, no lymph or adhesions were observed. There was a small blue spot on the surface of the bladder, on the left side, about the size of a half dime, through which the catheter perforated on taking hold of the bladder to feel for the broken instrument, and projected three fourths of an inch. On laying open the bladder, the coats were found thickened. The mucous membrane was sacculated, rough, and altogether quite morbid in its appearance. In the posterior part was a calculus formed round the middle of the catheter, both together weighing two and a half drachms. The piece of instrument, to which the calculus adhered firmly, was three and a half inches in length. The calculus was of a reddish-brown colour, extremely porous and friable. The prostate gland was about three times its natural size, and indurated.—*Ibid.*

*Comparative frequency of Phthisis in Man and in Animals.*—At the session of the Paris Academy of Sciences, on the 18th of July, M. RAYER read a very elaborate and interesting paper, entitled, "*Fragment of a Comparative Study of Phthisis Pulmonalis in Man and in Animals.*" The following are his conclusions:

1. Tuberculous Phthisis is of all chronic diseases the most common both among men and animals.

2. In man and other mammiferous animals, tuberculous matter may be readily distinguished from fresh pus, which always contains granular globules. In birds, the characters of tuberculous matter are less decidedly marked: the artificial introduction of foreign bodies into the lungs and muscles produces, not a white, opaque secretion, with granular globules, but a dry, yellowish substance, having no globules, the physical characters of which approach those of tubercles in the mammalia. In reptiles, fishes, and insects, the characters of tubercles are still less distinct.

3. Pus, in the mammalia, particularly in the horse, when deposited for a long period in the organs, undergoes successive transformations, which sometimes give it the appearance of tuberculous matter.

4. Pulmonary tubercules in man and quadrupeds, having a gray tint. In the lungs of the cow, tuberculous matter has usually a yellow chamois-leather colour.

5. In man and animals, the *central* softening of tubercles cannot be attributed to inflammation. It never presents globules of pus. The peripheric softening of tubercles is, on the contrary, most commonly promoted by inflammation of the surrounding tissues. It is almost always mixed with globules of pus.

6. The yellowish matter, which is found in the hydated cysts of ruminating animals after their rupture, has some analogy with the matter from the lungs; but the cysts, filled with this yellow matter, contain almost always the remains of the hydated sacs, and sometimes a certain amount of pus.

7. The cretaceous or calcareous concretions, composed chiefly of carbonate and phosphate of lime, which are seen in the lungs of men and animals, should not be considered, as has been heretofore the case, as nearly always a final modification of tubercle; they are often in man and very often in the horse, the remains of a small deposit of pus.

8. In many animals, there are formed in the lungs *verminous* granulations and *glanderous* granulations, which should be distinguished from *tuberculous* granulations.

9. In quadrupeds and in certain birds transported to temperate from warm climates, the development of phthisis has its maximum of frequency, almost to the exclusion of other chronic diseases. It is likewise promoted by a change of climate and of alimentation in other animals coming from the North, and particularly in the rein-deer.

10. Phthisis, which is rare in solipeds domesticated, is still more so in the carniverous animals. Nevertheless, in spite of the prophylactic influence of a strong constitution and animal diet, many carniverous animals, the domesticated cat, and, especially, the lion, and tiger, when transported into a temperate climate, may be attacked with pulmonary phthisis. This same infrequency of phthisis is found among birds, in birds of prey.

11. It is found that, of carniverous animals, the domesticated dog, of solipeds, the horse, are much less subject to tubercles than to cancer, a disease considered by Camper as unknown among animals.

12. In ruminating animals, particularly in the bovous tribe, phthisis is often found together with vesicular worms, particularly the echinocochia; but there is no foundation for the opinion that there is any connection of transformation or succession, between these hydatids and tubercles.

13. The fatty degeneration of the liver is generally a sign of phthisis in man and of general obesity in birds.

14. The alterations of the bones, which are observed in tuberculous monkeys, and particularly in those of America, appear analogous to the enlargement and spongy softening of the bones in phthisical and scrofulous children. Similar alterations are noticed in the bones of the carnivera of warm countries, transported into temperate latitudes.

15. While the frequency of pneumonia and the infrequency of phthisis, in the domestic dog, appear to indicate a want of connection between these two diseases, it is otherwise with the calf, the cow, and the milch ass, in which the deposit of tuberculous matter almost always coincides with a chronic progressive pneumonia.

16. Phthisis is hereditary, but it is almost never congenital even in the incipient stage.

17. In phthisical subjects, the sperm contained in the vesiculæ seminales has few or no spermatic animalcules.

18. Ulcers of the larynx, trachea, and bronchiæ, are not of the same import in man and in all animals. In the former, they almost always denote pulmonary phthisis and sometimes syphilis; in quadrupeds, a general tuberculous affection; in solipeds, almost always the glanders.

19. In pneumothorax, vegetations may be formed upon the pleura of a phthisical patient, as occurs sometimes in the air-cells of birds which are tuberculous or laboring under a lesion of the organs of respiration. In this case, as in all those noticed in the vertebrata, the development of this species of vegetations is always a *secondary* phenomenon.

From the foregoing conclusions, M. Rayer developed some general reflections, to which he called the attention of the Academy.

The progressive connection which anatomy and physiology demonstrate in the animal series, is shown also by pathology. It is owing to a parallelism of organization, that phthisis runs through so large a number of the vertebrata, until, as the scale of organization is lowered, the distinguishing characters of tubercles disappear and are not appreciable by our present means of investigation.

A predisposing cause in the production of tubercles in animals, is captivity or domestication; and, more comprehensively, a *decided and prolonged change in the natural state of existence.* The rein-deer coming from the North, the monkey from the South, both meet with the same end, when brought into captivity, although starting from opposite points. This cause, in intensity of action, may be compared to the bad lodging and nourishment, which, in man, so fearfully develop tuberculous phthisis.

Finally, in this vast series of tuberculous lesions, variable in their aspect, but ever the same, in animals of different natures, we see that phthisis is the common goal whither tend the various disturbances of the functions of nutrition, and that science, which, with regard to tubercles is powerless in curing, ought not to be powerless in preventing.—*Archives Générales de Médecine, August*, 1842.

COLLECTANEA MEDICA. *Abercrombie's Maxims for Medical Men.*

1. Cultivate a habit of steady and continuous attention.
2. Exercise strict control over the succession of your thoughts.
3. Keep up an animated, inquiring state of mind.
4. Maintain a habit of correct association of facts according to the relation of cause and effect.
5. Select carefully the subjects to which the mind is directed.
6. Carefully abstract the operation of the judgment from the influence of imagination or passion.

*Difficulties of the Medical Profession not to be considered as Discouragements.*—From the picture that has been exhibited of the innumerable doubts and difficulties which clog the attainment of medical knowledge, and embarrass the application of it to practical purposes, the timid, sceptical, and indolent may be discouraged from studies, apparently so arduous in their prosecution, and so questionable as to the efficiency and utility of their result. But it is not from characters of this description that any good can be expected in any of the useful arts of life.

If a like despondency were to pervade mankind in general, there would be an end to all that enterprise and energy which alone can enable them to act up to their destiny, and follow up those pursuits upon which the perfection of their nature depends.

As the senses would have lain dormant for ever had there been no external objects to stimulate them, so the faculties and virtues which characterise rational nature and civilised life would never have been developed, but through the excitement of those pains, wants, difficulties, and dangers inseparable from human life. By no other arrangement could our duties, our happiness, our mental and bodily perfections, have been bound together in one harmonious and consistent system.

Let us compare the art of medicine, under this aspect, with those of navigation and agriculture.

Had man been furnished by the Creator with wings, by which he could have traversed all seas and oceans, so as to supersede the use of ships, where would have been that hardihood of character and all those ingenious devices which have called forth the active energies and deep researches of the human mind?

If, contrary to the actual institutions of Providence, the life of man had been sustained by the spontaneous productions of nature, instead of the products of industry, neither the faculties of the mind nor the powers of the body could ever have been developed; man would have been little superior to the brutes; his active and inventive energies would have lain asleep for ever; there would have been no room for the talents exercised in the procuring of food, raiment and shelter, nor in commercial intercourse; all the mutual and endearing ties of social and civilised life; all trades and professions, arts and sciences, whether ministering to accommodation or elegance, constituting man's greatest felicity, whether as objects of pursuit or enjoyment, would have been unknown and untasted.—*Sir G. Blane's Medical Logic.*

*Anecdote flattering to the Medical Profession.*—But the anecdote most flattering to the medical profession which I should recal to your remembrance, is the occasion of the first establishment of the East India Company's power on the coast of Coromandel, which was procured by the favour of the

Great Mogul to one of our profession (Gabriel Boughton, of the ship Hopeful), in gratitude for his efficient help in a case of great distress to the monarch.

It seems that, in the year 1636 (a very early period of our direct intercourse with India), after the Portuguese had discovered the passage thither by the Cape of Good Hope, one of the princesses of the Great Mogul's family had been burnt dreadfully by accident, and that a messenger was sent to Surat, where foreign traders resorted, to desire the assistance of one of the English surgeons there, for they had acquired a great reputation among the natives for their skill in the cure of diseases. Gabriel Boughton proceeded forthwith to Delhi, and was successful in performing a cure; on which the Great Mogul's minister asked him what his master could do for him to manifest his gratitude for so important a service. Gabriel answered with a disinterestedness, a generosity, a patriotism beyond my praise, "Let my nation trade with yours." "Be it so," said the minister. A portion of the coast was marked out for the future resort of English ships, and all duties were compromised for a small sum of money. A better station, it is true, at the mouth of the Hoogly river, some twenty years afterwards, was chosen, and Calcutta was built; but here was the first establishment of our power—here did the civilisation of that vast continent begin. From hence the blessed light of the gospel may have been first promulgated amongst one hundred millions of native idolators, since subjected to British power, and made partakers of our enlightened comforts.—*Sir H. Halford, Medical Gazette.*

---

*Wound of the Antrum of Highmore, with destruction of the eye; fragment of knife-blade removed from the antrum two years afterwards.* By W. H. Donne, M. D., of Louisville. Reported by R. S. Wendel, Student of Medicine.—Schuti, a gardener, aged 42 years, a native of Germany, in a rencontre with an athletic man, on the 3d of May, 1840, was struck with a dirk-knife which entered about an inch above the right superciliary arch, passed through the corresponding eyelid, downwards and backwards, evacuating the humours of the eye, and penetrated the antrum. The globe of the eye was divided by a vertical incision, through which the aqueous humour escaped; the iris was extensively detached at the ciliary margin, and could be partially seen through the transparent cornea—its surface being somewhat obscured by small coagula. The hemorrhage was slight, and easily controlled by moderate pressure. The patient complained of intense pain in the temple and cheek of the wounded side, shooting deep into the orbit. Three points of interrupted suture were used to approximate the edges of the divided eye. Lint saturated with laudanum and warm water, constituted the dressing.

May 4th.—Some tumefaction in the eyelid; pulse 110; tongue coated and dry; skin hot; patient has spent a very restless night. Ordered following medicine: Tart. Emetic, gr. j.; Sulph. Magnesia, ℥ss.; to be dissolved in one-half pint of water, and a table-spoonful to be taken every half-hour, until nausea is induced—after which the interval may be increased.

May 5th—Bowels freely evacuated; pain less; skin moist; pulse 90 and soft. From this period until the wound healed—the space of three weeks—no constitutional symptoms of an untoward character occurred. The patient, however, contended that a portion of the knife-blade remained in the roof of his mouth. But on the most careful examination no foreign body could be detected.

On the 10th of August, 1842, Mr. Schuti called and requested Dr. Donne to examine his mouth, stating that for six months past he had been annoyed by a rough projecting substance, which some person had informed him was a piece of dead bone, but which he believed to be the point of the knife, that had been driven down into the bone by the violence of the blow. On looking into the mouth a small black speck was discernable about one-half inch from the interval between the first and second molar teeth. The parts adjacent were somewhat tumefied and inflamed. Dr. Donne made several attempts to extract this body with a pair of common dissecting forceps, but found it immoveably fixed in the substance of the bone. By dissecting around it with a bistoury down to the palate process of the superior maxillary bone, he was enabled to get a firmer hold, and with a pair of curved tooth forceps, succeeded in removing a fragment of the blade, $1\frac{1}{4}$ inches in length, and $\frac{3}{4}$ in. wide at the widest part; the extraction was not effected without considerable violence, and was attended with extreme suffering. The fragment came out with an audible snap, which induced those present to suppose at first that it had been broken; but on inspecting its surfaces closely, they were found similarly oxidised and wanting the lustre which a recent fracture would have presented. Upon probing the aperture through which the fragment had been extracted, no other piece could be detected. This opening would scarcely admit the curved probe which Dr. Donne passed into the antrum, in order to satisfy himself that the whole of the foreign body was removed. The next day there was a slight discharge from the aperture, though the patient has suffered very little pain since the operation.

[This case is certainly of a novel character. The rapidity with which the injury was repaired, and the length of time during which the fragment of the blade remained imbedded in the antrum, without causing much if any trouble to the patient, will at once strike the reader. It adds another to the many evidences on record of how much a sound constitution can effect, when aided by simple, yet prompt and judicious treatment such as was pursued by Dr. Donne in this instance. We should think it not improbable, however, that the patient may still have some difficulty, as the long sojourn of the broken knife in the antrum must have left the mucous membrane in a condition to take on disease from a slight cause.]—*Western Journal of Medicine and Surgery.*

---

*Respirators.*—Paragraphic notices have appeared, from time to time, in regard to the utility of respirators in this variable climate. Physicians are convinced of the utility of the invention, and the experience of invalids, which is worth infinitely more than any man's theory, confirms us in wishing that the instrument may be afforded at a price that will enable people of moderate circumstances to have it. Dr. Bowditch, of Bedford street, with this object in view, is about having respirators manufactured, equal in all respects to those brought from England, and that will be far more reasonable in cost. His advertisement will be found in to-day's Journal. Our medical friends, by keeping this intelligence in remembrance, can direct their patients where they can select from a large collection at a reasonable rate.—*Bost. Med. and Surg. Journ.* Oct. 5, 1842.

